# Infectious Esophagitis in Romanian Children: From Etiology and Risk Factors to Clinical Characteristics and Endoscopic Features

**DOI:** 10.3390/jcm9040939

**Published:** 2020-03-30

**Authors:** Mădălina Adriana Bordea, Alexandru Pîrvan, Dan Gheban, Ciprian Silaghi, Iulia Lupan, Gabriel Samașca, Lia Pepelea, Lia Monica Junie, Carmen Costache

**Affiliations:** 1 Department of Microbiology, IuliuHatieganu University of Medicine and Pharmacy, 400151 Cluj-Napoca, Romania; bordea_madalina@yahoo.com (M.A.B.); liapepelea@yahoo.com (L.P.); monicajunie@yahoo.com (L.M.J.); carmen_costache@yahoo.com (C.C.); 2 Department of Pediatrics II, IuliuHatieganu University of Medicine and Pharmacy, 400151 Cluj-Napoca, Romania; pirvanaaa@yahoo.com; 3 Department of Pathology, IuliuHatieganu University of Medicine and Pharmacy, 400151 Cluj-Napoca, Romania; dgheban@gmail.com; 4 Department of Biochemistry, IuliuHatieganu University of Medicine and Pharmacy, 400151 Cluj-Napoca, Romania; silaghiciprian@yahoo.com; 5 Department of Molecular Biology, Babes Bolyai University, 400151 Cluj-Napoca, Romania; iulia.lupan@gmail.com; 6 Department of Immunology, IuliuHatieganu University of Medicine and Pharmacy, 400151 Cluj-Napoca, Romania

**Keywords:** esophagitis, prevalence, risk factors, etiology, clinical characteristics, endoscopic assessment

## Abstract

Objectives. The aim of this study is to provide information about prevalence, etiology, risk factors, clinical characteristics and endoscopic features of various types of infectious esophagitis in children. Methods. We performed a total of 520 upper gastrointestinal tract endoscopies in Pediatric Clinic II, Emergency Hospital for Children, Cluj-Napoca. Indications for endoscopy in our cohort were gastrointestinal tract symptoms such as dysphagia, heartburn, or appetite loss. Results. The prevalence of infectious esophagitis in the study population was 2.11% (11 patients). Candida albicans (*C. albicans*) was the most frequent cause. Our data illustrates that herpes simplex virus (HSV)-induced esophagitis is common in immunocompromised patients and should be systematically suspected in cases of severe dysphagia, heartburn, or hematemesis. In the present study, all cytomegalovirus (CMV) esophagitis patients were immunocompromised. Immunodeficiency (81.8%) and prolonged antibiotic therapy with broad-spectrum antibiotics were by far the most important risk factors involved in the pathogenicity of the disease. Dysphagia, appetite loss, heartburn, epigastralgia, and hematemesis were the main clinical manifestations. Infectious esophagitis was associated with significant mortality. In four patients, endoscopy during life showed signs of infectious esophagitis; however, the precise etiology was only established post-mortem, in the pathological anatomy laboratory department. A risk factor involved in pathogenesis of post-mortem diagnosed infectious esophagitis is the DiGeorge syndrome for CMV and HSV patients. Conclusions. The study illustrates that infectious esophagitis should be considered in immunocompromised infants with prolonged antibiotic therapy with broad-spectrum antibiotics.

## 1. Introduction

Congenital or acquired immunodeficiency is related to a relatively increased prevalence of infectious esophagitis throughout the world [1]. Although increasing in prevalence in these particular population groups, until recently, only a few cases of infectious esophagitis have been reported in children in Eastern European countries. According to various studies, there are many risk factors that can predispose to infectious esophagitis, including the systemic autoimmune diseases, acquired immunodeficiency syndrome (AIDS), antibiotics and steroids use, chemotherapy, radiotherapy, malignancies, organ transplantation, and malnutrition [2,3,4].

Infectious esophagitis can be produced by bacteria, fungi, viruses, or parasites. Candida albicans (*C.albicans*) is by far the most common cause of infectious esophagitis, followed by Cytomegalovirus (CMV), herpes simplex virus (HSV) and the Epstein–Barr virus (EBV) [2]. *C. albicans*-induced esophagitis is common in immunosuppressed patients.

*C albicans*, the most prevalent fungal species of the human microbiota, can attain a dominant position in the gut flora soon after long-term antibiotic therapy. The most frequent agent of candidiasis is *C.albicans*, although other species are occasionally involved [3]. Once *C.albicans* has colonized the gut, it can be difficult to reduce the proportion of Candida spp. This is due to the way that it creates biofilm that is intrinsically resistant to conventional antifungal therapeutics and the host immune system [4].

Infectious esophagitis more often affects immunocompromised patients, but it can occasionally be seen in immunocompetent hosts with different types of comorbidities such as achalasia, gastric metaplasia in the esophagus, or scleroderma. Published data suggest that HSV esophagitis can occur in apparently immunocompetent individuals, while CMV esophagitis is well-documented in immunocompromised patients [5,6,7,8].

The aim of this retrospective study is to identify the prevalence, etiology, risk factors, clinical characteristics, and endoscopic features of various types of infectious esophagitis in children in our geographic area (Romania, Cluj county). Because the majority of infectious esophagitis occurring in immunocompetent hosts have fewer, less-specific symptoms, these conditions are probably underdiagnosed at the present time [9,10,11].

## 2. Material and Methods

We performed a retrospective analysis of the endoscopic biopsies database records of pediatric patients (under 18 years old). Patients were diagnosed with different types of gastrointestinal disorders, and gastroscopies were performed at a single center in Cluj-Napoca. From January 2000 to December 2014, a total of 520 upper gastroscopies were performed in Pediatric Clinic II, Emergency Hospital for Children, Cluj-Napoca.

All study subjects underwent upper gastrointestinal endoscopies to confirm the clinical diagnosis. Indications for endoscopy in our cohort were gastrointestinal tract symptoms such as dysphagia, heartburn, hematemesis, vomiting, or appetite loss. Biopsies were performed in our study population because gastroscopies revealed characteristic endoscopic findings of various types of infectious esophagitis (*C.albicans*, CMV, HSV-induced esophagitis). Written informed consent was obtained from all patients and their parents. The exclusion criteria were patients with coagulation dysfunctions or psychiatric diseases. Hematoxylin–eosin (HE) and periodic acid–Schiff (PAS) staining, followed by cultures and histologic examination, were used to identify etiology. Culture is the gold standard for diagnosis. Viral esophagitis was diagnosed based on the presence of typical cytopathic epithelial changes in the esophageal biopsy specimens. Cytopathological examination of esophageal brushings showed cellular changes consistent with HSV and CMV infection [6,7,8,11]. Viral culturefrom esophageal biopsy was positive for HSV-1and CMV. Histologic confirmation of *C.albicans* in the esophagus was the gold standard for diagnosis. HE stain of biopsies or brushing of esophageal candidiasis showed pseudohyphae. Cultures return positive for Candida. Although tissue immunohistochemistry and PCR would have been alternate methods to confirm the etiology, due to technical difficulties, we were not able to perform these assays in our clinic.

The study was approved by the Ethical Committee of the IuliuHatieganu University of Medicine and Pharmacy Cluj-Napoca.

## 3. Results

### 3.1. Prevalence and Etiology

The prevalence of infectious esophagitis in this retrospective cohort study was 2.11% (11/520 patients). Nine patients out of eleven (81.8%) were immunosuppressed (*p* < 0.01), and two were immunocompetent patients without comorbidities. In four patients, endoscopy during life showed signs of infectious esophagitis; however, the precise etiology was only established post-mortem in the pathological anatomy laboratory department. *C.albicans* caused the majority of endoscopy-confirmed infectious esophagitis (45.45%, 5/11), followed by CMV (36.36%, 4/11), and HSV (18.19%, 2/11). It is important to mention that CMV was involved in two cases of post-mortem diagnosed infectious esophagitis, followed by HSV and *C.albicans* in equal percentages. Demographic data and predictors for infectious esophagitis were analyzed. There were five girls (45.45%) and six boys (54.55%). The median age was 6.04 years. The peak incidence of HSV esophagitis was at two years of age, with stomatitis as clinical first expression. Only two patients had a recent history of prolonged antibiotic therapy.

### 3.2. Risk Factors and Laboratory Diagnosis

Blood testing for CMV and HSV type 1 Immunoglobulin G and Immunoglobulin M were positive in our study population. In five patients with endoscopically proven esophagitis, immunological tests, including total immunoglobulins titer and T-lymphocytes subsets, were abnormal. Nephrotic syndrome and leukemia were the major causes of immunosuppression in our study population. Risk factors and comorbidities involved in the pathogenesis of post-mortem diagnosed infectious esophagitis was the DiGeorge syndrome for CMV and HSV-induced esophagitis. The only case of post-mortem diagnosed *C.albicans* esophagitis was in connection with HIV infection (Table 1). CMV infection involved the entire GI tract, lungs, and liver for our post-mortem diagnosed CMV-induced esophagitis patients. The causes of death in the DiGeorge syndrome patients were heart failure and arrhythmia. Pneumonia and toxic shock syndrome were the most common causes associated with death in HIV-infected patients.

There were two patients with no evidence of any immune deficiency. Therefore, we report two cases of endoscopically-diagnosed *C.albicans* esophagitis in immunocompetent hosts without comorbidities but with anamnestic evidence of prolonged antibiotic therapy with broad spectrum antibiotics for acute pneumonia.

### 3.3. Clinical Characteristics

The most common gastrointestinal symptoms were dysphagia, heartburn, appetite loss, epigastralgia, hematemesis, fever, and nausea. Dysphagia (45%) and appetite loss (22%) were the most common clinical manifestations in *C.albicans*-induced esophagitis. Heartburn (48%), dysphagia (24%), and hematemesis (12%) were the most frequently symptoms in patients with HSV esophagitis, while dysphagia (52%), followed by epigastralgia (34%) and severe gastrointestinal bleeding (9%) were the main clinical manifestations in patients with CMV esophagitis. The presence of oral candidiasis and evidence of herpetic lesions on the lips were identified only in a few patients (two patients with *C.albicans* and one patient with HSV esophagitis).

### 3.4. Endoscopic Assessment

**Figure 1:**
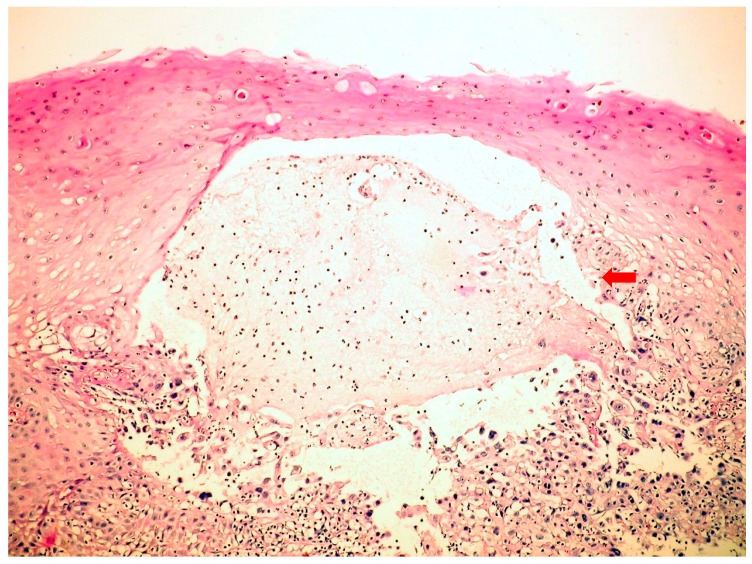
Herpes simplex virus (HSV)-inducedesophagitis (microscopy-HE, 400×) with serous vesicle formation.

**Figure 2:**
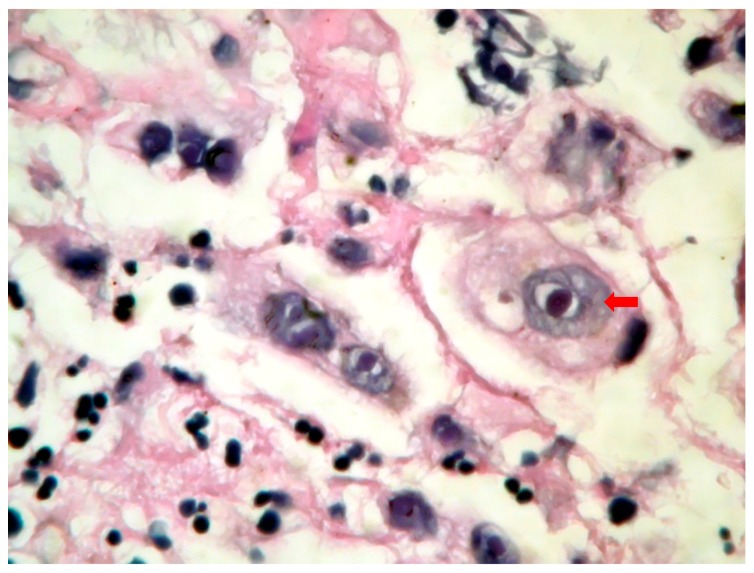
Cytomegalovirus (CMV)-induced esophagitis (microscopy-HE, 1000×):intracytoplasmic and intranuclear large, basophilic inclusion bodies surrounded by a clear halo (Cowdry type B).

The endoscopic features were quite characteristic. Upper gastrointestinal endoscopy in *C.albicans* esophagitis revealed multiple, elevated, adherent, and confluent white plaques localized in the upper, mid, and lower esophagus. The esophageal mucosa had a friable and erythematous appearance. Stomach and duodenum were normal. To confirm diagnosis, biopsies were performed, showing the presence of yeasts and pseudohyphae into mucosal cells. Microbiologic cultures of brushings obtained from the esophagus were positive for *C.albicans*. Esophagogastroduodenoscopy in HSV esophagitis showed multiple, superficial, small, well-circumscribed, vesicular “punched-out” ulcers with acute inflammation and normal intervening mucosa, starting from the mid and extending to lower esophagus. In patients with post-mortem-diagnosed IE, lesions were spread throughout the entire esophagus. Biopsies taken from the edge of these ulcers revealed typical cytopathic epithelial changes, including multinucleated giant cells and intranuclear eosinophilic inclusion bodies. Cytological examination of the esophageal biopsy specimens showed an ulcerated esophageal mucosa (Figure 1) with acute inflammation and intranuclear and intracytoplasmic characteristics inclusion bodies (Cowdry type A). Esophagogastroduodenoscopy in CMV esophagitis revealed a single, large, and deep ulcer, located in the middle section of the esophagus. A severe form of esophagitis with increased friability was also found in this kind of infection. Cytology smears from esophageal brushings revealed an ulcerated esophageal mucosa with acute inflammation and intracytoplasmic and intranuclear large (Figure 2), basophilic inclusion bodies (PAS +) surrounded by a clear halo (Cowdry type B). Antifungal therapy with Fluconazole was initiated in *C.albicans*-induced esophagitis patients, and, within two weeks after starting therapy, symptoms were reduced. Acyclovir therapy was used in HSV-induced esophagitis patients, while Ganciclovir was required for treatment in CMV-induced esophagitis patients. A second endoscopy at the end of the fungal or viral treatment was not performed.

## 4. Discussion

*C.albicans*-induced esophagitis is a common condition seen particularly in children and older patients treated with antibiotics, chemotherapy, or radiotherapy. This etiology of infectious esophagitis is also frequent in patients with hematologic malignancies. Children treated with inhaled steroids, such as those with asthma and those with immune deficiency, such as AIDS, are also at high risk for *C.albicans* infectious esophagitis [2]. We report only two cases of endoscopically-diagnosed *C.albicans* esophagitis in immunocompetent hosts without comorbidities. For the two cases of *C.albicans*-induced esophagitis in immunocompetent hosts, reported in our study, there is evidence of prolonged antibiotic therapy with broad-spectrum antibiotics, based on anamnesis. The only case of post-mortem-diagnosed *C.albicans* esophagitis was in connection with HIV infection. Our data can only suggest that *C.albicans*-induced esophagitis is more common in immunosuppressed patients than in immunocompetent hosts. This was similar to other reports in which most of the patients were immunocompromised or with a long history of antibiotics use [9,12,13,14,15]. Different studies have shown that heartburn and epigastralgia are the most common symptoms in *C.albicans* infectious esophagitis; however, Jae Hyeuk Choi et al. showed that more than half of the patients were asymptomatic [3]. Our data illustrates that dysphagia and appetite loss can be considered as common symptoms in *C.albicans*-induced esophagitis patients, as these were the most frequently mentioned complains of our pediatric patients [1,2]. Gastrointestinal endoscopy in *C.albicans*-induced esophagitis patients showed multiple raised white plaques throughout the entire esophagus. These results are in line with previous studies [9,10,11,12].

HSV-induced esophagitis is a severe infection that occasionally appears in immunocompetent hosts [16,17,18]. All of our patients with HSV-induced esophagitis were immunocompromised. The disease is mostly related to HSV type 1. HSV type 2-induced esophagitis has rarely been reported [10,11,16]. All our patients had disease caused by HSV type 1. Previous data suggest that patients treated with inhaled steroids are at high risk. Reactivation of herpetic infection with the spread of the virus to the esophagus by direct extension from the oral cavity can appear following a course of low dose corticotherapy and can induce HSV esophagitis. Few case reports of HSV reactivation associated with corticotherapy have been published [18]. In our data, as well as in the majority of cases reported, HSV-induced esophagitis occurred in the absence of corticotherapy. Herpetic esophagitis is usually self-limited in immunocompetent patients. Primary HSV infection is common in childhood. By adolescence, 90% of all individuals possess antibodies to Type 1 HSV [2,6], as well as in our cohort. The most common clinical presentation includes acute onset of dysphagia, heartburn, and fever. Our patients also present dysphagia and heartburn; nevertheless, the third common symptom was hematemesis instead of fever. Endoscopy in HSV esophagitis revealed multiple superficial, small, well-circumscribed, vesicular ulcers with acute inflammation and normal intervening mucosa in the distal and mid esophagus. Endoscopic findings were similar to those found in the literature for patients with endoscopy-confirmed esophagitis [6,11,17]. Therefore, the classic predominance of lesions in the lower half of the esophagus was only observed in patients with endoscopy-confirmed infectious esophagitis. In patients with post-mortem diagnosed infectious esophagitis, lesions were spread throughout the entire esophagus.

According to different studies, CMV-induced esophagitis is well-documented in immunocompromised patients [2,5,7,8]. One of the largest studies is from Wang et al. [5]. The immunocompromised conditions most frequently reported by Wang as being associated with CMV-induced esophagitis are AIDS, followed by bone marrow transplantation, autoimmune diseases, and malignancies. On the other hand, Bonetti et al. [19] have shown, in a case series of 30 patients with CMV-induced esophagitis, that 50% of them were immunocompetent hosts. In the present study, all four patients with CMV esophagitis were immunocompromised. Even though in our study the patients were symptomatic, the majority of immunocompetent hosts are less symptomatic, which may explain the underdiagnosis. Similar to previous studies, our research can emphasize that dysphagia, followed by epigastralgia and hematemesis, were common symptoms [2,5,7]. Earlier data on this topic showed that distal ulcers are more common than mucosal inflammation [7,19,20]. In general, ulcers seen in CMV-induced esophagitis tend to be large and deep, and are more likely to be found in the distal section of the esophagus. However, in our data, both mid-esophageal mucosa ulceration and acute inflammation were observed in all cases. Our results are in contrast with many previous clinical studies in adults and children [2,5,7,19,20,21].

A risk factor involved in the pathogenesis of post-mortem-diagnosed infectious esophagitis was the DiGeorge syndrome for CMV and HSV patients. Post-mortem-diagnosed *C.albicans* esophagitis was in connection with HIV infection. CMV infection is usually associated with a wide range of complications, includinghepatitis, pneumonia, gastritis, and colitis [21]. It is important to note that CMV infection involved the entire gastrointestinal tract, lungs, and liver for our post-mortem diagnosed CMV-induced esophagitis patient. Our data suggest that infectious esophagitis, as well as different other pathologies of the esophagus [22], is a rare and underdiagnosed pathology in children.

## 5. Conclusions

The study illustrates that infectious esophagitis should be considered in immunocompromised infants with prolonged antibiotic therapy with broad-spectrum antibiotics, with *C.albicans* as the main etiologic agent of endoscopy-confirmed infectious esophagitis. Our data illustrates that HSV-induced esophagitis is common in immunocompromised patients and should be systematically suspected in cases of severe dysphagia, heartburn, or hematemesis. HSV-induced esophagitis is a severe life-threatening disease with a wide variety of symptoms. CMV infection involved all parts of the gastrointestinal tract in patients with post-mortem-diagnosedinfectious esophagitis. The esophagus was the most affected site by the infection. CMV-induced esophagitis was diagnosed late and was associated with severe complications such as severe hematemesis and death. Our data highlights that both mid-esophageal ulceration and acute inflammation are the most common endoscopic features.

Infectious esophagitis can be associated with significant mortality. It is a serious disease (four of our patients were diagnosed only post-mortem) but with a relatively low population prevalence. Ante-mortem diagnosis was frequently missed in the early stages of infection including in *C.albicans*-induced esophagitis. Therefore, early use of endoscopy in patients with an unidentified cause of odynophagia, dysphagia, or heartburn (gastroesophageal reflux disease symptoms) is required for diagnosis. Prevalence may be underestimated, especially in less symptomatic immunocompetent hosts. Although the number of gastroscopies analyzed was 520, only 11 (2.11%) presented infectious esophagitis. Until the present time, limited pediatric data, including some case reports, involved viral esophagitis as an important cause of mortality and morbidity in Romania and Eastern European countries.

## Figures and Tables

**Table 1:** Etiology/risk factors/comorbidities.

Diagnostic Method	Etiology	Number of Patients	Risk Factors/Comorbidities
Endoscopy	CMV	2	Immunodeficiency of unknown cause
Post-mortem	CMV	2	Immunodeficiency—DiGeorge syndrome
Endoscopy	HSV	1	Immunodeficiency of unknown cause
Post-mortem	HSV	1	Immunodeficiency—DiGeorge syndrome
Endoscopy	*C.albicans*	2	Immunodeficiency of unknown cause
Endoscopy	*C.albicans*	2	Prolonged antibiotic therapy with broad-spectrum antibiotics
Post-mortem	*C.albicans*	1	Immunodeficiency—HIV infection
